# Decreased Endogenous Nitric Oxide Production in Patients with Acute Decompensated Heart Failure with Preserved Ejection Fraction

**DOI:** 10.3390/jcm14175928

**Published:** 2025-08-22

**Authors:** Roman Falls, Bing H. Wang, Sara Vogrin, Christopher J. Neil

**Affiliations:** 1 School of Health and Biomedical Sciences, RMIT University, Melbourne, VIC 3083, Australia; 2 Baker Heart and Diabetes Institute, and Monash Alfred Baker Centre for Cardiovascular Research, Monash University, Melbourne, VIC 3004, Australia; 3 Department of Medicine, University of Melbourne-Western Health, Melbourne, VIC 3010, Australia

**Keywords:** nitric oxide, plasma nitrite, heart failure, asymmetric dimethylarginine, arterial stiffness

## Abstract

**Background:** Heart failure with a preserved ejection fraction (HFpEF) accounts for approximately 50% of patients with heart failure. Endothelial dysfunction has been documented in HFpEF, and impaired nitric oxide (NO) production may be a contributing factor in patients decompensating from chronic to acute HFpEF. Plasma nitrite (NO_2_^−^), but not plasma nitrate (NO_3_^−^), is highly reflective of local nitric oxide production. However, plasma NO_2_^−^ levels in relation to chronic and acute HFpEF patients have not been studied. **Methods:** Plasma NO_2_^−^ and NO_3_^−^ concentrations were quantified with gas-phase chemiluminescence. Plasma asymmetric dimethylarginine (ADMA), an endogenous inhibitor of NO production, and arterial stiffness were also quantified. Samples were collected from 19 participants with chronic HFpEF and 16 participants with acute HFpEF. **Results:** Plasma NO_2_^−^ concentrations were lower in participants with acute HFpEF when compared to the chronic HFpEF group (*p* = 0.022). NO_3_^−^, ADMA and indices of arterial stiffness did not display any significant between-group differences. **Conclusions:** We present novel NO_2_^−^ data, which has not been previously quantified in patients with acute HFpEF. Our results indicate that plasma concentrations of NO_2_^−^ may be decreased in patients with acute HFpEF compared to patients with chronic HFpEF, suggesting a dysregulated NO pathway. Further studies are required to confirm these findings in this patient population.

## 1. Introduction

Approximately 50% of patients who receive a heart failure diagnosis, are diagnosed with heart failure with preserved ejection fraction (HFpEF) [1]. HFpEF is typically characterised by concentric remodelling of the left ventricle (LV), impaired LV relaxation whilst maintaining a left ventricular ejection fraction (LVEF) greater than 50% [2]. Current evidence suggests that HFpEF is more prevalent in females than males, skewed towards older age, and more likely to have associated systolic hypertension [3]. Current epidemiological evidence indicates that the prevalence of HFpEF is increasing in both absolute terms and also relative to heart failure with reduced ejection fraction (HFrEF) [1]. The increasing prevalence of HFpEF is thought to be due to various factors, such as an ageing population, increased prevalence of metabolic syndromes (i.e., diabetes mellitus), hypertension, and obesity [4].

Despite some recent advances in HFpEF treatment, such as SGLT-2 inhibition [5,6], prognostic data in patients with HFpEF remains poor, with reported 5-year mortality rates as high as 75% [7]. Further exploration into the mechanisms of the pathophysiology of the syndrome and what constitutes decompensation is warranted in this population.

Endothelial dysfunction has been documented in patients with HFpEF [8,9]. Aging, obesity, hypertension, dyslipidaemia, diabetes, and cigarette smoking, all risk factors for HFpEF, have been associated with poor endothelial function [10,11]. Previous studies have suggested that endothelial dysfunction and arterial stiffness may play a central role in HFpEF, both in its onset and decompensation [12,13].

Nitric oxide (NO) is a key regulatory molecule in the vasculature that is produced in endothelial cells. Catalysed by endothelial nitric oxide synthase (eNOS), NO diffuses into adjacent vascular smooth muscle cells and produces vasodilation through the activation of soluble guanylate cyclase. The production of NO from eNOS is decreased by asymmetric dimethylarginine (ADMA), an endogenous inhibitor of eNOS. The pathophysiology of endothelial dysfunction regarding NO and ADMA has been well established in various population groups, but is yet to be fully characterised in HFpEF.

Significant difficulties exist pertaining to quantifying NO concentrations in vivo due to its very short half-life (milliseconds). NO is rapidly oxidised to plasma nitrite (NO_2_^−^) and nitrate (NO_3_^−^), which have been used as surrogate markers of NO. There is much evidence to suggest that plasma NO_2_^−^, and not plasma NO_3_^−^, better reflects NO production from eNOS, largely due to the high background concentration of plasma NO_3_^−^ [14,15,16]. Thus, this study aimed to explore the pathophysiology of HFpEF related to indices of NO biology, in both compensated and decompensated states by exploring plasma concentrations of NO_2_^−^ and ADMA and their relationships with augmentation index (AIx), an indicator of arterial stiffness, in patients with acute decompensated and chronic HFpEF.

## 2. Methods

### 2.1. Study Design and Protocol

This cross-sectional study enrolled participants prospectively into either the chronic HFpEF group or the acute decompensated HFpEF group. In order to more truly represent the wider population with HFpEF, the inclusion and exclusion criteria were deliberately kept broad (see Table 1). All participants in this study were recruited from Sunshine Hospital (Western Health), either from the Heart Failure Clinical (Chronic group) or from the Coronary Care Unit (Acute group). All participants had an LVEF > 50% and evidence of elevated LV filling pressures on echocardiography.

The Melbourne Health Human Research Committee approved this study (HREC/18/MH/250, approved 22 June 2018). This study was also approved by the Research Governance Office at Western Health (2018.170). Participation in this study was explained to all eligible participants during a screening visit. Those who agreed to participate signed a participant information consent form prior to commencement.

### 2.2. Aims and Hypotheses

Evidence from the literature indicates that NO_2_^−^ and not NO_3_^−^ is reflective of local NO production [14,15,16]. NO_2_^−^ has not been quantified in patients with acute HFpEF. Given that NO is a potent vasodilator, and that NO_2_^−^ is indicative of local NO production, further investigation in patients with acute and chronic HFpEF is warranted. As such, the aim of this study was to quantify NO_2_^−^ and NO_3_^−^, in addition to ADMA and AIx, in patients with acute decompensated and chronic HFpEF. Our primary hypothesis was that participants with acute decompensated HFpEF would exhibit lower concentrations of plasma NO_2_^−^ compared to participants with chronic HFpEF. Our secondary hypotheses were that plasma ADMA concentrations and AIx would be higher in patients with acute decompensated HFpEF compared to those with chronic HFpEF. Additionally, we also hypothesised that there would be an inverse correlation between plasma NO_2_^−^ concentrations and both plasma ADMA concentrations and AIx, regardless of HF phase.

### 2.3. Study Measures

An NOA-280i nitric oxide analyser from Sievers Instruments (Boulder, CO, USA) was used to determine NO_2_^−^ and NO_3_^−^ concentrations in plasma. This NO analyser utilises gas-phase chemiluminescence to determine the quantity of these nitrogen-species, the methodology for which has been previously described [17]. Standard curves of known quantities of sodium NO_2_^−^ (0–500 nM) and sodium NO_3_^−^ (0–1000 µM) were generated both prior to and after batch sample analysis, using a protocol that has previously been described [18]. ADMA concentrations were quantified utilising a human ADMA ELISA kit from My BioSource (MBS264847, San Diego, CA, USA). All samples for this analysis were analysed in duplicates and were reported in µM. The ADMA detection range was 0.078–5.0 µM with a sensitivity of 0.01 µM; all results were read at 450 nm. AIx was determined utilising a SphygmoCor XCEL device (AtCor Medical, West Ryde, NSW, Australia). A specialised sphygmomanometer cuff was placed in the standard position on the brachial artery to determine AIx in each participant. AIx is calculated by this device as the ratio of augmentation pressure to pulse pressure. Participants were asked to remain still and seated throughout the data acquisition process. B-type natriuretic peptide (BNP) plasma concentrations were quantified using a human ELISA kit (Abcam, Cambridge, UK, ab193694).

### 2.4. Statistical Analysis

GraphPad Prism version 9.0.1 for macOS (GraphPad Software, San Diego, CA, USA) and Statistical Package for the Social Sciences (version 22 (SPSS Inc., Chicago, IL, USA) were used to perform the statistical analysis. The set a-priori statistical significance level was ≤0.05. All figures were created using GraphPad Prism v 9.0.1. For parametric data, between-group differences were tested utilising unpaired *t*-tests. Conversely, a Mann-Whitney U test was performed for non-parametric data. Pearson’s chi-squared test or Fisher’s exact test (where appropriate) was performed on categorical baseline characteristics. Due to a lack of published NOA data in the acute phase of HFpEF in the literature, nitric oxide metabolites (NO_x_) data were used from Saitoh et al. (2003) [19].

Covariates for the multivariable statistical model were determined by utilising a binary backwards likelihood ratio logistic regression. All categorical variables are presented as n (%). We have previously published similar works in patients with chronic and acute decompensated heart failure with a reduced ejection fraction (HFrEF) [20]. The data from this HFrEF cohort were used to compare with data from the current HFpEF cohort. Participants in the acute decompensated HFrEF group were prescribed more loop diuretics and had a higher prevalence of hypertension.

## 3. Results

Baseline characteristics are reported in Table 2. Thirty-five participants with HFpEF were recruited in this study, 19 with chronic HFpEF and 16 with acute decompensated HFpEF. All participants had an ejection fraction ≥ 50% as per inclusion criteria (see Table 1). More loop diuretics and fewer angiotensin receptor blockers were prescribed to participants in the acute HFpEF group. Mean systolic blood pressure, was higher in the chronic HFpEF group. E/e′ and pulmonary capillary wedge pressure were higher in the acute decompensated group; see Table 2.

### 3.1. Nitric Oxide Analysis in Chronic and Acute Phases of HFpEF

Plasma NO_2_^−^ was lower in patients with acute HFpEF compared to patients with chronic HFpEF (*p* = 0.022); see Table 3. Plasma NO_3_^−^ did not display a statistically significant difference between groups (*p* = 0.23). Neither plasma NO_2_^−^ nor NO_3_^−^ displayed significant between-group differences after adjusting for smoking status and anti-coagulant use. In the total HFpEF population, plasma NO_2_^−^ did not exhibit a significant correlation with respect to plasma NO_3_^−^ (r_s_ = 0.12, *p* = 0.56).

**Figure 1:**
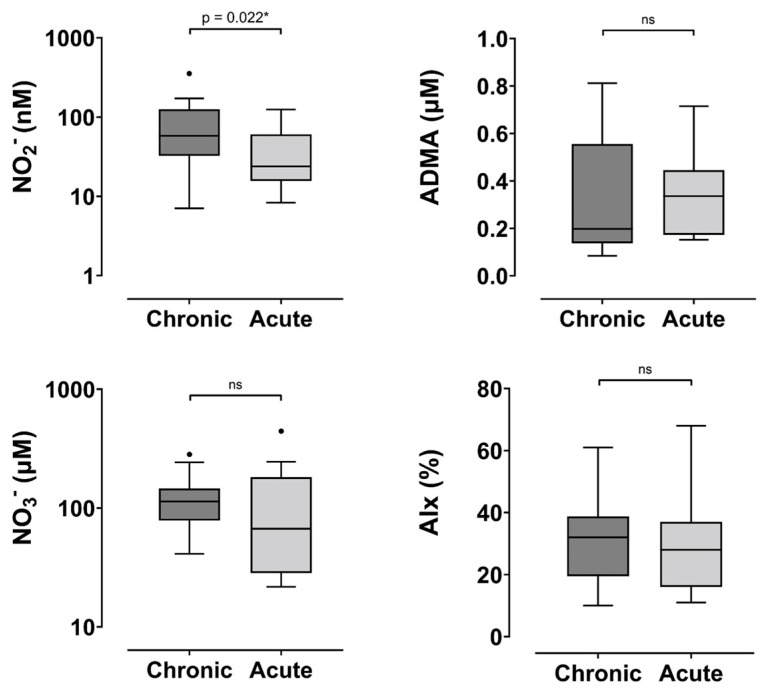
Comparison of outcome measures in chronic and acute HFpEF. Abbreviations: NO_2_^−^—nitrite, NO_3_^−^—nitrate, ADMA—asymmetric dimethylarginine, AIx—augmentation index. * indicates *p* < 0.005, ns—not significant.

Despite a median ADMA being 1.9 times higher in chronic compared to the acute phase of HFpEF, no statistically significant difference between groups was observed in plasma ADMA concentrations (*p* = 0.34) (Figure 1). No statistically significant difference was observed in AIx in patients with chronic and acute HFpEF (*p* = 0.75). In contrast to our secondary hypotheses, plasma NO_2_^−^ did not display any significant correlations with NO_3_^−^, ADMA, or AIx in the entire cohort of patients with HFpEF.

### 3.2. Comparison Between HF Phenotypes

Our group has previously published a similar analysis of NO_2_^−^, NO_3_^−^, ADMA, and AIx in patients with HFrEF [20]. In the analysis below, we compared these variables between the HFrEF and HFpEF phenotypes, with direct comparisons made between the same phase in each cohort.

Analysis of study measures was performed comparing the chronic phase of HFrEF to the chronic phase of HFpEF. No significant difference was observed in plasma NO_2_^−^ concentrations between the phenotypes of HF (*p* = 0.99). Similarly, no significant difference was observed with respect to plasma NO_3_^−^ concentrations between the chronic subtypes of HF (*p* = 0.90). No statistically significant differences were observed in AIx values and plasma ADMA concentrations (*p*-values = 0.248 and 0.163, respectively).

Analysis of study measures was performed comparing the acute phase of HFrEF to the acute phase of HFpEF. There was no observed, statistically significant difference in plasma NO_2_^−^ concentrations between the two subtypes of HF in the acute phase (*p* = 0.76). Similarly, no statistically significant difference was observed regarding plasma NO_3_^−^ concentrations in the acute phase of HF (*p* = 0.89). No statistically significant differences were observed in AIx values and plasma ADMA concentrations (*p*-values = 0.73 and 0.20, respectively); see Table 4.

## 4. Discussion

For the first time, we present novel and preliminary plasma NO_2_^−^ and NO_3_^−^ data in patients with acute and chronic HFpEF. Lower plasma NO_2_^−^ concentrations were observed in patients with acute HFpEF (*p* = 0.022). Previously published data in healthy individuals reported basal plasma NO_2_^−^ levels at approximately the 300 nM level [15,21]. In the absence of a healthy control group, this may be taken to suggest that plasma NO_2_^−^ concentrations in both our sample groups are markedly reduced compared to healthy populations. Furthermore, patients in the acute phase of HFpEF appear to exhibit a more marked reduction in plasma NO_2_^−^ concentrations relative to patients with chronic HFpEF. Previous studies have demonstrated in animal and human populations that plasma NO_2_^−^ is highly reflective of NO production via eNOS [15,16,22]. Hence, these data may be taken to suggest that endothelial dysfunction is prevalent in patients with HFpEF and may worsen as patients become decompensated with clinical congestion. It should be noted that despite achieving statistical significance on a univariate analysis, NO_2_^−^ concentrations were not significant between groups after adjusting for covariates in the results we have presented. Consequently, these results are limited in their clinical utility but may play an important role in hypothesis generation for future research.

### Comparison of NOA Findings with Previous Literature

To our knowledge, only one study has quantified plasma NO_2_^−^ levels in patients with chronic HFpEF utilising gas-phase chemiluminescence. Ratchford et al. (2019) found mean baseline concentrations of plasma NO_2_^−^ of 182 nM in a clinical trial investigating various antioxidants in patients with chronic HFpEF [23]. Eggebeen et al. (2016) [24] found mean plasma NO_2_^−^ levels in patients with chronic HFpEF to be 340 nM. This was analysed with a variant of the Griess method [24]. Other authors have attempted to measure plasma NO_2_^−^ in patients with chronic HFpEF using varying techniques. In a trial investigating the impact of infusion sodium NO_2_^−^ on exercise haemodynamic and ventricular performance, plasma NO_2_^−^ levels were undetectable before infusion [25]. Plasma NO_2_^−^ was quantified utilising a liquid chromatography-fluorometric assay, which is described in more detail by Rix et al. (2015) [26]. Assay sensitivity data was not reported. Similar findings were also reported by the same group in patients with HFpEF prior to inhaled sodium NO_2_^−^ [27]. Plasma NO_2_^−^ levels were not measured in the INDIE-HFpEF trial [28]. To the best of our knowledge, no studies have quantified NO_2_^−^ or NO_3_^−^ in patients with acute HFpEF. Interestingly, plasma NO_3_^−^ and plasma NO*_x_* concentrations exhibited no statistically significant difference between chronic and acute HFpEF groups (*p* = 0.23). Plasma NO_3_^−^ exists in much larger (>1000-fold) background concentrations compared to plasma NO_2_^−^ and is more influenced by factors such as dietary nitrate consumption. Previous studies have shown that plasma NO_2_^−^ but not plasma NO_3_^−^, is reflective of local endothelial-derived NO [14,15,16]. Despite this, NO_3_^−^ and/or NO_x_ are still commonly measured as indices of endothelial NO production. Our findings of significantly lower plasma NO_2_^−^ but no significant difference in plasma NO_3_^−^ concentrations, further highlight the importance of measuring plasma NO_2_^−^ as an inference of endothelial NO production.

Despite plasma median ADMA concentrations in the acute HFpEF group being almost double that compared to the chronic HFpEF group, no between-group difference was observed (*p* = 0.338). Plasma ADMA concentrations have been reported by other groups with chronic HFpEF [29] and have been shown to be elevated in patients with HFpEF compared to healthy controls [30]. Furthermore, this same study by Hage et al. (2020) [30] found increases in the arginine to ADMA ratio, MPO and calprotectin in patients with HFpEF compared to healthy controls. Participant numbers in our study were low and it is plausible that a significant difference may have been observed if the participant numbers were increased. 

No significant differences were observed in AIx between chronic and acute HFpEF groups (*p* = 0.75). Furthermore, AIx was not elevated in either of our allocated groups, yielding similar findings to previous reports in healthy subjects [31]. Evidence suggests that arterial stiffness can be reduced by various common HF therapeutics that act against the neurohumoral axis [32,33,34,35]. It is possible that in patients with HFpEF, who are often concomitantly taking many of these therapeutic agents known to decrease indices of arterial stiffness, the measurement of arterial stiffness may be underestimated. Further investigation in this area is needed to support this claim.

## 5. Limitations

This study was powered for differences in plasma NO_x_, as no plasma NO_2_^−^ data were available in acute HFpEF in the literature. Due to the cross-sectional design of this study, no causative conclusions from lower NO_2_^−^ concentrations in acute decompensated HFpEF can be drawn. The lack of an age-matched healthy control group also limits the generalisability of the results and controlling for confounding variables. This study would have also benefited from increased participant numbers. Finally, many medications, such as statins and renin-angiotensin-aldosterone system blockers, can independently affect endothelial function. Future research should include greater participant numbers and a healthy control group, so that further sub-analysis of the impact of typical heart failure medications can be performed.

## 6. Conclusions

In summary, this is the first time plasma NO_2_^−^ has been quantified and compared in patients with acute and chronic HFpEF. These preliminary findings suggest that patients with acute HFpEF exhibit significantly lower plasma NO_2_^−^ concentrations compared to their chronically compensated counterparts, whilst simultaneously displaying no significant difference in plasma NO_3_^−^ concentrations. These results appear to indicate impaired endothelial function and decreased NO bioavailability in patients with acute HFpEF. Further research with a larger sample size is required to confirm these findings.

## Figures and Tables

**Table 1:** Inclusion and exclusion criteria.

	Inclusion Criteria	Exclusion Criteria
Chronic HFpEF group	≥18 years of age Confirmed diagnosis of chronic HF as per the Framingham criteria Ejection fraction ≥ 50%	Currently being treated in a palliative manner or being referred to palliative care Presence of a significant cognitive impairment
Acute decompensated HFpEF group	≥18 years of age Confirmed diagnosis of ADHF as per the Boston criteria Ejection fraction ≥ 50%	Currently being treated in a palliative manner or being referred to palliative care Presence of a significant cognitive impairment

**Table 2:** Baseline characteristics of HFpEF study population.

Variable	Chronic HFpEF (n = 19)	Acute HFpEF (n = 16)	*p*-Value
Male	8 (42)	10 (63)	0.23
Age	69 ± 15	68 ± 11	0.87
**Co-morbidities**			
Hypercholesterolaemia	12 (63)	8 (50)	0.43
Hypertension	13 (68)	13 (81)	0.39
IHD	9 (47)	8 (50)	0.88
Stroke	1 (5)	3 (19)	0.31
AF	8 (42)	11 (69)	0.12
Diabetes mellitus	8 (42)	11 (69)	0.12
Smoker	10 (53)	4 (25)	0.096
Ex-Smoker	6 (32)	10 (63)	0.067
Current	3 (16)	2 (13)	1.0
Alcohol abuse	1 (5)	0 (0)	1.0
Substance abuse	1 (5)	0 (0)	1.0
Thyroid disease	2 (11)	5 (31)	0.21
Iron deficiency/Anaemia	3 (16)	4 (25)	0.66
OSA	4 (21)	7 (44)	0.27
Gout	2 (11)	2 (13)	1.0
COPD/Asthma	5 (26)	4 (25)	1.0
GORD	4 (21)	3 (19)	1.0
Cancer	0 (0)	2 (13)	0.2
CKD	4 (21)	4 (25)	1.0
**Medications**			
Beta Blocker	16 (84)	13 (81)	0.82
ACEi	9 (47)	8 (50)	0.88
ARB	6 (32)	0 (0)	0.022 *
Organic nitrate	6 (32)	2 (13)	0.24
CCB	2 (11)	2 (13)	1.0
Lipid lowering agents	15 (79)	10 (63)	0.28
OHA	8 (42)	8 (50)	0.64
Insulin	3 (16)	7 (44)	0.13
Loop diuretic	14 (74)	16 (100)	0.027 *
Thiazide diuretic	2 (11)	2 (13)	1.0
MRA	9 (47)	11 (69)	0.2
Digoxin	2 (11)	1 (6)	1.0
PPI	5 (26)	6 (38)	0.48
Aspirin	9 (47)	8 (50)	0.87
Other Anti-platelets	4 (21)	3 (19)	1.0
Anti-coagulant	8 (42)	11 (69)	0.12
Sacubitril/Valsartan	1 (5)	0 (0)	1.0
**Echocardiographic and haemodynamic variables**			
LVEF (%)	55 ± 5	58 ± 8	0.16
RAP (mmHg)	5.5 ± 3.5	6.9 ± 4.4	0.25
RVSP (mmHg)	33.1 ± 14.1	40.6 ± 8.8	0.063
LAVI (ml/m^2^)	42.5 ± 13.6	43.2 ± 15.8	0.92
E/e′	16 (12.5–21.5)	20 (17–27)	0.04 *
PCWP (mmHg) ^#^	21.74 (17.4–28.6)	26.7 (23.0–35.4)	0.04 *
sysBP (mmHg)	147.0 ± 18.3	132.2 ± 18.1	0.026 *
diaBP (mmHg)	79.1 ± 13.3	75.2 ± 13.8	0.42
ADP (mmHg)	85.5 ±16.5	76.7 ± 13.8	0.11
MAP (mmHg)	100.1 ± 13.9	93.8 ± 14.0	0.21
HR (bpm)	65.4 ±10.8	71.8 ± 10.7	0.1
ASP (mmHg)	133.1 ± 15.1	120.0 ± 14.9	0.018 *
APP (mmHg)	52.8 ± 14.6	43.3 ± 13.7	0.066
BNP	2105 (1155–3055)	1603 (1080–3546)	0.65

Abbreviations: IHD—ischemic heart disease, AF—atrial fibrillation, OSA—obstructive sleep apnoea, COPD—chronic obstructive pulmonary disease, GORD—gastro-oesophageal reflux disease, CKD—chronic kidney disease, ACEi—angiotensin-converting enzyme inhibitor, ARB—angiotensin 2 receptor blocker, CCB—calcium channel blocker, OHA—oral hypoglycaemic agent, MRA—mineralocorticoid receptor antagonist, PPI—proton pump inhibitor, HFpEF—heart failure with a preserved ejection fraction, LVEF—left ventricular ejection fraction, RAP—right atrial pressure, RVSP—right ventricular systolic pressure, LAVI—left atrial volume index, PCWP—pulmonary capillary wedge pressure, sysBP—systolic blood pressure, diaBP—diastolic blood pressure, MAP—mean arterial pressure, HR—heart rate, ASP—aortic systolic pressure, ADP—aortic diastolic pressure, APP—aortic pulse pressure, BNP—B-type natriuretic peptide. Categorical variables are presented as n (%). Normally distributed continuous variables are presented as mean ± standard deviation. Non-normally distributed data are presented as median (interquartile range). ^#^ PCWP was non-invasively derived from Nagueh’s formula; * indicates *p*-value < 0.05.

**Table 3:** Analysis of study measures in chronic and acute HFpEF.

Variable	Chronic HFpEF	Acute HFpEF	*p*-Value ^1^	OR (95% CI)	*p*-Value ^2^
NO_2_^−^ (nM)	58.1 (32.5–125.4)	23.9 (15.7–60.4)	0.022 *	0.98 (0.96–1.0)	0.077
NO_3_^−^ (µM)	113 (78.4–145.9)	66.9 (28.4–181.7)	0.23	1.0 (0.99–1.0)	0.80
ADMA (µM)	0.19 (0.14–0.55)	0.37 (0.17–0.45)	0.34	0.93 (0.63–1.36)	0.68 ^3^
AIx ^†^	30.6 ± 13.4	29 ± 15	0.75	0.99 (0.94–1.05)	0.77

Abbreviations: HFpEF—heart failure with a preserved ejection, NO_2_^−^—plasma nitrite, NO_3_^−^—plasma nitrate, ADMA—asymmetric dimethylarginine, AIx—augmentation index, OR—odds ratio, 95% CI—95% confidence interval. Unless otherwise indicated, data are non-parametric and are reported as median (interquartile range). Ex-smoker and current anti-coagulant use were both significant in the final step of the backwards elimination logistic regression and were considered covariates in the multivariable analysis; ^†^ indicates normally distributed data and is reported as mean ± standard deviation; ^1^ indicates *p*-values from the univariate analysis; ^2^ indicates *p*-values from the multivariable analysis; ^3^ odds ratio for 0.1 increase in ADMA; * indicates *p*-value < 0.05.

**Table 4:** Comparison of study measures between HF phenotypes in each phase of HF.

Variable	HFpEF	HFrEF	*p*-Value
**Chronic Phase**			
NO_2_^−^ (nM)	58.1 (32.5–125.4)	60 (30.4–209.3)	0.99
NO_3_^−^ (µM)	113 (78.4–145.9)	96.7 (82.9–153.2)	0.89
ADMA (µM)	0.19 (0.14–0.55)	0.16 (0.13–0.24)	0.16
AIx (%) ^†^	30.6 +/− 13.4	25.6 (12.0)	0.25
**Acute Phase**			
NO_2_^−^ (nM)	23.9 (15.7–60.4)	30.7 (19.1–52.4)	0.76
NO_3_^−^ (µM)	66.9 (28.4–181.7)	63.3 (36.9–161.8)	0.89
ADMA (µM)	0.37 (0.17–0.45)	0.4 (0.27–0.56)	0.20
AIx (%) ^†^	29 ± 15	26.7 (23.3)	0.73

Abbreviations: NO_2_^−^—plasma nitrite, NO_3_^−^—plasma nitrate, ADMA—asymmetric dimethylarginine, AIx—augmentation index, HFpEF—heart failure with a preserved ejection fraction, HFrEF—heart failure with a reduced ejection fraction. Unless otherwise indicated, data are non-parametric and are reported as median (interquartile range); ^†^ indicates normally distributed data, reported as mean ± standard deviation.

## Data Availability

The data presented in this study are available upon request from the corresponding author.
